# Feasibility, Acceptability and Early Outcomes of Concomitant Aortic and Mitral Valve Surgery via a Single-Incision Right Anterior Minithoracotomy: A Retrospective Cohort Study

**DOI:** 10.3390/jcm15166159

**Published:** 2026-08-08

**Authors:** Lukman Amanov, Sadeq Ali-Hasan-Al-Saegh, Arian Arjomandi Rad, Jawad Salman, Fabio Ius, Stefan Rümke, Khalil Aburahma, Jan Dieter Schmitto, Bastian Schmack, Arjang Ruhparwar, Alina Zubarevich, Alexander Weymann

**Affiliations:** 1 Department of Cardiothoracic, Transplantation and Vascular Surgery, Hannover Medical School, Carl-Neuberg-Straße 1, 30625 Hannover, Germany; 2 Department of Cardiothoracic Surgery, Oxford Heart Centre, John Radcliffe Hospital, Oxford University Hospitals NHS Foundation Trust, Oxford OX3 9DU, UK

**Keywords:** aortic valve surgery, mitral valve surgery, concomitant, minithoracotomy

## Abstract

**Background**: Minimally invasive approaches for multivalve surgery have attracted increasing interest; however, data on combined aortic and mitral valve replacement or repair using via right anterior minithoracotomy remain quite limited. This study aimed to evaluate the feasibility, safety, and early outcomes of minimally invasive concomitant aortic and mitral valve replacement or repair using this approach. **Methods**: This retrospective study included 24 patients who underwent simultaneous aortic and mitral valve procedures via right anterior minithoracotomy. We collected preoperative, intraoperative, and postoperative data, assessing echocardiographic parameters. Early clinical outcomes, complications, and mortality rates were analyzed, with correlations between EuroSCORE II and outcomes explored. **Results**: The median follow-up was 412 days. All procedures were completed successfully without conversion to sternotomy. Postoperative echocardiography demonstrated a significant reduction in transvalvular gradients, with aortic mean pressure gradient decreasing from 51.3 ± 23.0 mmHg to 6.7 ± 1.7 mmHg (*p* < 0.001) and mitral mean pressure gradient from 19.3 ± 26.7 mmHg to 4.0 ± 1.4 mmHg (*p* < 0.001), while left ventricular ejection fraction remained unchanged (*p* = 0.67). During the study period, one patient died from a non-cardiac cause. EuroSCORE II showed a moderate positive correlation with intensive care unit length of stay (*p* = 0.011) but not with hospital stay or operative times. **Conclusions**: Minimally invasive aortic and mitral valve replacement or repair via right anterior minithoracotomy is feasible and was associated with favorable early hemodynamic and clinical outcomes in this single-center cohort.

## 1. Introduction

The surgical management of patients with concomitant aortic and mitral valve disease represents a significant therapeutic challenge. While conventional median sternotomy provides broad operative exposure and is considered the gold standard, it often results in considerable surgical trauma. This increased trauma can lead to significant postoperative pain, a higher incidence of sternal complications, extended recovery times, and unsatisfactory cosmetic outcomes, particularly in elderly and comorbid patients [1,2].

To address the need for reduced surgical morbidity, minimally invasive cardiac surgery (MICS) has progressed over the last two decades from a novel approach to an accepted standard for isolated mitral and tricuspid valve procedures in specialized medical centers [3]. Right anterior minithoracotomy (RAT) is increasingly used for mitral valve surgery and has been associated with favorable clinical outcomes, including reduced blood loss, shorter hospital stays, and improved patient satisfaction in appropriately selected patients [4]. RAT has been established as a standardized, reproducible, and minimally invasive approach for mitral valve surgery, facilitating femoral cannulation and providing excellent surgical access [5]. In contrast, the adoption of surgical aortic valve replacement (SAVR) through RAT has remained limited among cardiac surgeons, primarily because of its greater technical complexity and the associated steep learning curve [6].

Despite these advances, performing concomitant aortic and mitral valve surgery through a single minimally invasive access remains technically demanding. This approach requires substantial surgical expertise to overcome the challenges of limited operative exposure, complex de-airing maneuvers, and the execution of two major valvular procedures within a restricted surgical field. As a result, the available evidence remains limited and is largely confined to small case series and technical reports [7,8]. The limited availability of robust evidence regarding the standardized feasibility, perioperative risk profile, and hemodynamic performance of this approach represents an important gap in the current literature, hindering the development of evidence-based surgical strategies for patients with complex multivalvular heart disease. Recent reports have further expanded the evidence supporting the feasibility of minimally invasive multivalve surgery for the treatment of complex valvular heart disease [9,10].

In this study, we report our single-center experience with a consecutive cohort of patients who underwent concomitant biological aortic and mitral valve surgery through RAT approach. The primary objective was to evaluate the feasibility and early outcomes of this minimally invasive strategy. Secondary objectives included the assessment of hemodynamic outcomes by systematic echocardiographic evaluation and the characterization of postoperative recovery and early clinical outcomes.

## 2. Materials and Methods

### 2.1. Study Design and Patient Selection

We conducted a retrospective, single-center cohort study including 24 consecutive patients who underwent concomitant aortic and mitral valve replacement or repair through a right anterior minithoracotomy between August 2023 and December 2025. Eligibility for the minimally invasive approach was determined through multidisciplinary discussion within the cardiac surgery team, taking into account anatomical suitability, clinical characteristics, and procedural complexity. Patients requiring conventional median sternotomy because of anatomical or clinical considerations were treated accordingly and were not included in the present study. Individuals who underwent isolated valve procedures, required alternative surgical access, needed concomitant coronary bypass, or underwent full sternotomy were excluded from the study. All operations within the defined study period were reviewed.

### 2.2. Ethical Statement

As per local German regulations, ethical approval was not mandatory for this study due to its retrospective design and non-interventional nature.

### 2.3. Surgical Technique

All operations were performed by a single surgical team. Under general anesthesia with endotracheal intubation, patients were positioned with the right side elevated 30°. All procedures were performed under direct vision through the right anterior minithoracotomy without endoscopic visualization. A 4–5 cm incision was made in the third intercostal space along the right midclavicular line. Following systemic heparinization and achievement of an adequate activated clotting time, cardiopulmonary bypass (CPB) was established via the femoral vessels. A long venous cannula was advanced through the femoral vein into the superior vena cava under echocardiographic guidance. Arterial cannulation was subsequently performed via the femoral artery, and cardiopulmonary bypass was initiated under normothermic conditions. Aortic occlusion was achieved with a Chitwood clamp introduced through a separate port. Myocardial protection was achieved through a single administration of 2000 mL of antegrade Custodiol^®^ histidine–tryptophan–ketoglutarate (HTK) cardioplegia (Koehler Chemie, Alsbach-Hähnlein, Germany) directly into the aortic root. If ischemic time exceeds 120 min, a repeated dose is administered. The left atrium was opened parallel to the interatrial groove. The mitral valve replacement or repair was performed first, followed by the aortic valve procedure via a transverse aortotomy. In cases using a sutureless aortic bioprosthesis, the aortotomy was minimized. After de-airing, the aorta and left atrium were closed. All patients received biological prostheses. Protamine was administered to reverse systemic heparinization after the termination of CPB. Chest tubes were placed as needed, and the minithoracotomy was closed in layers.

### 2.4. Data Acquisition

Demographic and clinical characteristics were extracted from the institutional cardiac surgery registry and electronic medical records. Preoperative variables included age, sex, body mass index, body surface area, renal function indices, cardiovascular risk factors, history of chronic obstructive pulmonary disease (COPD), atrial fibrillation status (paroxysmal or chronic), presence of cardiac implantable devices, and history of endocarditis. EuroSCORE II was calculated for all patients. Intraoperative data included operative duration, aortic cross-clamp and cardiopulmonary bypass times, type and frequency of cardioplegia administration and transfusion requirements.

Postoperative outcomes were evaluated, including duration of ventilation, length of stay in the intensive care unit, total hospital stay, and a comprehensive assessment of adverse events such as new-onset atrial fibrillation, third-degree atrioventricular (AV) block, conduction disturbances necessitating pacemaker implantation, major bleeding, reoperation, conversion to conventional full sternotomy, respiratory insufficiency, need for tracheostomy, renal failure requiring dialysis, wound complications, sepsis, neurological deficits, ischemic stroke, seizures, delirium, intracranial bleeding, vascular injury, moderate-to-severe paravalvular leakage, structural valve deterioration, myocardial infarction, and the need for mechanical circulatory support (ECLS/ECMO/Impella).

### 2.5. Outcome Definitions

Paravalvular leak (PVL) refers to any gap or channel between the anatomical annulus and the prosthetic valve, which leads to a regurgitation jet flowing between two heart chambers. Structural valve deterioration describes the intrinsic, acquired changes within the prosthesis, such as leaflet calcification, tears, fractures of the stent-frame, and damage to other components of the valve. Early mortality was defined as death occurring either during the index hospital stay or within 30 days post-surgery.

### 2.6. Echocardiographic Assessment

Routine intraoperative transesophageal echocardiography was performed in all patients to evaluate the immediate surgical result and to exclude significant residual valvular regurgitation or paravalvular leakage. Transthoracic echocardiography (TTE) was routinely performed preoperatively and prior to hospital discharge. Key echocardiographic parameters collected included left ventricular ejection fraction (LVEF), severity of valvular regurgitation and stenosis, transvalvular gradients, aortic peak velocity, and systolic pulmonary artery pressure.

### 2.7. Statistical Analysis

All analyses were conducted using SPSS version 28 (IBM Corp., Armonk, NY, USA) and R software (version 4.1.1, R Foundation for Statistical Computing, Vienna, Austria). Continuous variables underwent normality testing using the Shapiro–Wilk method. Normally distributed variables were reported as mean ± standard deviation; non-normally distributed variables were expressed as median with interquartile range. Categorical variables were summarized as absolute and relative frequencies. For Perceval sutureless valves (Corcym SRL, Saluggia, Italy, previously LivaNova), labeled sizes (S, M, L, XL) were converted to nominal annular diameters in millimeters according to the manufacturer’s specifications to allow quantitative analysis. The upper limit of the recommended annular range was used for statistical calculations. Paired comparisons between preoperative and postoperative echocardiographic parameters were conducted using paired *t*-tests for normally distributed variables and Wilcoxon signed-rank tests for non-normally distributed variables. Associations between EuroSCORE II and continuous clinical outcomes such as ICU and hospital length of stay were assessed using Spearman rank correlation due to non-normal distributions and limited sample size. A two-tailed *p*-value < 0.05 was considered statistically significant.

## 3. Results

A total of 24 patients were included in the study. The median follow-up was 412 days. The mean age was 72.0 ± 5.9 years, and 9 patients (37.5%) were female. Mean body weight was 75.6 ± 21.1 kg, with a mean body mass index of 24.5 ± 5.2 kg/m^2^ and a mean body surface area of 1.9 ± 0.2 m^2^. The mean EuroSCORE II was 3.1 ± 2.2%. Arterial hypertension was present in 20 patients (83.3%), hyperlipidemia in 12 (50.0%), and atrial fibrillation in 14 (58.3%), including chronic atrial fibrillation in 5 patients (20.8%) and paroxysmal atrial fibrillation in 9 patients (37.5%). Chronic kidney disease and chronic obstructive pulmonary disease were each present in 5 patients (20.8%), whereas insulin-dependent diabetes mellitus was observed in 2 patients (8.3%). Infective endocarditis was present in 3 patients (12.5%) (Table 1).

The mean preoperative LVEF was 51.3 ± 11.2%. Systolic pulmonary artery pressure was 32.3 ± 7.4 mmHg, with a median of 30.0 mmHg (IQR, 26.5–35.0 mmHg). Preoperative valvular assessment demonstrated advanced disease severity, with significant aortic stenosis identified in 19 patients (79%), aortic regurgitation in 7 patients (29%), and mitral regurgitation in 20 patients (83%). The mean preoperative aortic mean pressure gradient was 51.3 ± 23.0 mmHg, and mitral mean pressure gradient was 19.3 ± 26.7 mmHg (Table 2). Predischarge TTE demonstrated no patient with mild or greater residual mitral regurgitation or clinically relevant paravalvular leakage. When present, residual mitral regurgitation was limited to physiologic or trace regurgitation without hemodynamic significance.

All procedures were successfully completed via RAT, with no conversions to sternotomy. The mean operative time was 252.6 ± 47.58 min, CPB duration 159.6 ± 33.8 min, and cross-clamp time 109.6 ± 26.8 min. A single cardioplegia dose was administered in the majority of patients. Additional concomitant procedures beyond aortic and mitral valve surgery were performed in a minority of patients. Left atrial appendage closure was undertaken in 4 patients (16.7%), whereas no patient underwent concomitant MAZE ablation (Table 3).

In the aortic position, biological prostheses were implanted in all patients. The most frequently used valve types included Perceval (Corcym SRL, Saluggia, Italy, previously LivaNova), Hancock II (Medtronic, Minneapolis, MN, USA), Epic (St. Jude Medical/Abbott, St. Paul, MN, USA) and Inspiris (Edwards Lifesciences, Irvine, CA, USA) prostheses. The mean aortic prosthesis size was 24.2 ± 1.0 mm, with sizes ranging from 23 to 25 mm. In the mitral position, mitral valve replacement was performed in 13 patients (54%), whereas mitral valve repair using annuloplasty rings was achieved in 11 patients (46%). For mitral valve replacement, biological prostheses were used in all cases, with a mean valve size of 28.2 ± 1.6 mm (range 27–31 mm). Mitral valve repair was carried out using Memo 4D annuloplasty rings (Corcym, Saluggia, Italy), with a mean ring size of 33.5 ± 2.0 mm (range 32–36 mm). No mechanical prostheses were implanted (Table 4).

Postoperative outcomes were favorable overall (Table 5). The median intensive care unit stay was 2 days (IQR, 1–3 days) and the median hospital stay was 11 days (IQR, 9–15 days). No cases of new-onset atrial fibrillation were observed, whereas high-grade atrioventricular block requiring permanent pacemaker implantation occurred in 2 patients (8.4%). Re-thoracotomy for major bleeding was required in 1 patient (4.2%), postoperative renal replacement therapy in 2 patients (8.4%), and respiratory insufficiency requiring prolonged ventilatory support in 1 patient (4.2%). No postoperative stroke occurred. No cases of wound dehiscence or surgical site infection were observed during the postoperative period. No patient required postoperative mechanical circulatory support with extracorporeal membrane oxygenation (ECMO) or Impella^®^ device implantation. In-hospital and 30-day mortality was 4.2% (1 patient). The death was unrelated to the cardiac procedure and resulted from severe gastrointestinal bleeding in a patient with a known history of gastrointestinal disease despite repeated endoscopic hemostatic interventions.

Postoperative echocardiography demonstrated excellent prosthetic valve performance (Table 2). The aortic MPG decreased markedly from 51.3 ± 23.0 mmHg preoperatively to 6.7 ± 1.7 mmHg postoperatively (*p* < 0.001). Similarly, mitral MPG decreased from 19.3 ± 26.7 mmHg to 4.0 ± 1.4 mmHg (*p* < 0.001). LVEF remained stable (preoperative 51.3 ± 11.2% vs. postoperative 51.9 ± 10.7%, *p* = 0.67). No structural valve deterioration or significant postoperative prosthetic regurgitation was observed in either valve position.

A statistically significant moderate positive correlation was observed between EuroSCORE II and intensive care unit length of stay (*p* = 0.011), indicating that patients with higher preoperative risk scores required prolonged postoperative intensive care. In contrast, no significant correlations were identified between EuroSCORE II and overall hospital length of stay (ρ = 0.10, *p* = 0.63), cardiopulmonary bypass duration (ρ = 0.08, *p* = 0.68), or aortic cross-clamp time (ρ = 0.05, *p* = 0.78).

## 4. Discussion

The present study demonstrates that simultaneous aortic and mitral valve replacement or repair through a single right anterior minithoracotomy is both technically feasible and clinically safe in a carefully selected patient population. In our cohort of 24 consecutive patients, all procedures were successfully completed through a single minimal-access incision without conversion to sternotomy or intraoperative mortality. The procedural success rate of 100% and 30-day mortality rate of 4.2% highlight that even complex bivalvular pathologies can be addressed safely through RAT when it is performed by an experienced surgical team.

### 4.1. Summary and Interpretation of Our Results

Our cohort represented a typical elderly population with multiple comorbidities. Despite this elevated risk profile (median EuroSCORE II 3.16%), operative and postoperative outcomes were favorable. There was no significant difference in operative time; however, cardiopulmonary bypass and cross-clamping times in anterolateral thoracotomy group were significantly longer than those reported in the literature for the standard anterior thoracotomy group [11].

Cardioplegia was administered as a single dose using Custodiol (Koehler Chemie, Alsbach-Hähnlein, Germany) (2000 mL), providing homogeneous myocardial protection. Postoperative LVEF remained stable (51.3% preoperatively vs. 51.9% postoperatively), confirming that myocardial protection was adequate. There were no cases of postoperative myocardial infarction or low-output syndrome. This finding is consistent with the work of Pisano et al., who demonstrated that intra-aortic occlusion and antegrade cardioplegia can be safely achieved through a right anterior minithoracotomy without compromising myocardial preservation [5].

Importantly, concomitant procedures such as left atrial appendage closure were successfully performed in several patients (16.7%). These results confirm that RAT provides sufficient exposure for both the mitral and aortic valves while permitting additional atrial interventions. Sharma et al. similarly demonstrated that MAZE and LAA procedures can be incorporated into RAT mitral surgery with excellent safety [12]. The ability to perform such adjunctive operations without conversion underscores the versatility of this approach.

No conversions to sternotomy occurred in our series, which compares favorably with reported conversion rates of up to 3% in other minimally invasive or redo valve surgery series [13]. In addition, there were no vascular complications, groin site injuries, or limb ischemia. Guedes et al. previously reported that this technique reduces peripheral vascular risks while maintaining excellent surgical exposure [8].

### 4.2. Hemodynamic and Echocardiographic Outcomes

Postoperative echocardiography confirmed excellent prosthetic valve function and hemodynamic performance. The mean aortic pressure gradient decreased dramatically from 51.3 ± 23.0 mmHg preoperatively to 6.7 ± 1.7 mmHg postoperatively (*p* < 0.001), and the mitral gradient from 19.3 ± 26.7 mmHg to 4.0 ± 1.4 mmHg (*p* < 0.001). These reductions are consistent with prior findings from large series of minimally invasive AVR and MVR, which demonstrate that the limited exposure of a thoracotomy does not compromise prosthetic function or positioning [7,8].

No cases of paravalvular leak or structural valve deterioration were observed, and no reoperations for valve dysfunction were necessary during hospitalization or at early follow-up. Ahmad et al. similarly reported excellent early outcomes in totally endoscopic combined valve replacement using automated annular suturing systems, with no leaks or prosthesis failures [14]. The absence of such complications in our cohort supports the precision and reproducibility of RAT-AMVR in experienced hands.

### 4.3. Postoperative Course

The postoperative course in our series was characterized by rapid recovery and low morbidity. The median ICU stay was 2 days, and total hospital stay 11 days, all of which compare favorably with contemporary minimally invasive valve programs [15].

Permanent pacemaker implantation was required in two patients (8.4%), consistent with prior data indicating that minimally invasive approaches do not increase conduction-related complications relative to sternotomy [16,17].

Early complications were rare: re-thoracotomy for major bleeding was required in only one case (4.2%), and there were no strokes, myocardial infarctions, or wound-healing problems. No cases of mediastinitis, pneumothorax, or sternal dehiscence occurred, reflecting one of the main advantages of thoracotomy over sternotomy. These findings compare favorably with conventional combined valve replacement, where neurological complications occur in up to 8% and wound complications in up to 6% [18].

At a median follow-up of 412 days, there were no cases of prosthetic degeneration or dysfunction, demonstrating early durability comparable to the long-term results of Glauber et al. in more than 1600 minimally invasive mitral procedures [3]. Furthermore, we observed a statistically significant correlation between EuroSCORE II and ICU length of stay (ρ = 0.51, *p* = 0.011), indicating that patient preoperative risk—rather than access route—remains the dominant determinant of recovery. Similar observations were made by van Kampen et al. in their multicenter analysis of minimally invasive mitral repair programs [19].

### 4.4. Technical Considerations and Implications

The success of RAT-AMVR relies on several critical technical factors. Our standardized sequence—mitral valve replacement or repair first via left atriotomy, followed by transverse aortotomy for the aortic valve—proved reproducible and efficient. This sequence allows stable exposure, minimizes interference between prostheses, and facilitates complete de-airing before closure. Pitsis et al. described a comparable procedural sequence in totally endoscopic AMVR, achieving excellent hemodynamic outcomes and short cross-clamp times [20].

Ahmad et al. [14] conducted a study from September 2020 to June 2023 involving 66 consecutive patients (mean age 61.8 ± 11 years) who underwent endoscopic minimally invasive aortic or mitral valve replacement via RAT at two cardiac surgery referral centers in Germany. In this cohort, isolated aortic valve surgeries accounted for 81.8%, isolated mitral valve surgeries for 15.2%, and only one case (1.5%) involved simultaneous aortic and mitral valve surgery. Our report specifically focuses on the simultaneous aortic and mitral valve surgeries performed through a right anterior minithoracotomy in 24 cases. Thus, our report represents one of the largest sample sizes to date for this specific procedure [14].

The flexibility to choose between central or peripheral femoral cannulation remains a significant advantage of this technique [8,11,12]. Additionally, the RAT incision provides direct visualization of both valve annuli and facilitates safe manipulation of prosthetic sewing rings, even in complex anatomy.

The ability to perform concomitant MAZE and LAA closure procedures highlights the flexibility of this approach. Botta et al. demonstrated that such combined interventions can be safely integrated into minimally invasive programs without prolonging bypass time or increasing morbidity [15]. In our experience, the addition of these procedures was technically straightforward and did not significantly affect operative duration.

Our findings are in agreement with those reported by Zoni et al. [10], who described a series of 105 patients undergoing totally endoscopic aortic valve replacement with or without concomitant major cardiac procedures, including 57 patients treated with concomitant aortic and mitral valve disease. Their study demonstrated that combined valve procedures could be performed safely through the same endoscopic working port without increasing in-hospital mortality or postoperative stroke. Although their surgical strategy relied on a totally endoscopic approach, whereas our procedures were performed through a direct-vision right anterior minithoracotomy, both studies demonstrate that minimally invasive concomitant aortic and mitral valve surgery can be performed safely and effectively in appropriately selected patients [10].

The role of minimally invasive double-valve surgery should also be considered within the expanding transcatheter treatment landscape. Transcatheter mitral valve implantation (TMVI) may represent an alternative for selected patients at prohibitive or high surgical risk, including those undergoing valve-in-valve, valve-in-ring, or valve-in-mitral annular calcification procedures. However, TMVI remains technically challenging because of the need for comprehensive multimodality imaging, patient-specific anatomical assessment, device-specific access and anchoring requirements, paravalvular regurgitation, valve thrombosis, and particularly the risk of left ventricular outflow tract obstruction. Therefore, treatment selection should be individualized through multidisciplinary Heart Team discussion, balancing clinical characteristics, anatomical suitability, procedural complexity, and the anticipated risks and benefits of surgical and transcatheter approaches [21].

### 4.5. Study Limitations and Future Perspectives

This study is limited by its retrospective, single-center design and relatively small sample size. Nonetheless, it provides one of the few focused analyses of true combined aortic and mitral valve replacement or repair via a single RAT incision using standardized methodology. The absence of conversion to sternotomy, the low rate of early postoperative complications, and the favorable short-term hemodynamic outcomes observed in this cohort support the technical feasibility of this approach.

Future research should include larger multicenter studies to assess long-term prosthetic durability, particularly of biological valves implanted via this route. Moreover, the incorporation of robotic assistance, 3D visualization, and automated suture devices may further simplify exposure and shorten the learning curve for surgeons adopting this technique [14,15,16,17,18].

The future of complex valve surgery is likely to involve hybrid or fully endoscopic strategies, combining the safety of surgical exposure with the reduced invasiveness of transcatheter technologies. Early reports suggest that hybrid minimally invasive AMVR and transcatheter reinterventions could become acceptable alternatives in high-risk or redo populations [19,22,23,24].

## 5. Conclusions

Simultaneous aortic and mitral valve replacement or repair through a single right anterior minithoracotomy is a technically demanding but reproducible procedure. In our series, it was associated with excellent hemodynamic correction, preserved ventricular function, and minimal morbidity. This approach facilitates additional procedures such as MAZE ablation, left atrial appendage closure, surgical treatment of ascending aortic aneurysms, aortic root surgeries, bypass for significantly stenosing right coronary arteries, and tricuspid valve repair, all without compromising exposure or increasing risk. With growing institutional experience and evolving instrumentation, RAT-AMVR may represent a feasible minimally invasive approach for carefully selected patients with concomitant aortic and mitral valve disease.

## Figures and Tables

**Table 1:** Comprehensive Demographic Characteristics.

Variables	
Age	72.0 ± 5.9
Weight	75.6 ± 21.1
Body Mass Index, kg/m^2^	24.5 ± 5.2
Body Surface Area, m^2^	1.9 ± 0.2
Creatinine	63.6 ± 28.2
Glomerular Filtration Rate	72.5 ± 20.1
EuroSCORE II, %	3.1 ± 2.2
Female sex	9 (37.5%)
Arterial hypertension	20 (83.3%)
Hyperlipidemia	12 (50.0%)
Coronary artery disease (CAD)	2 (8.3%)
History of PCI	2 (8.3%)
History of myocardial infarction < 90 days	2 (8.3%)
Chronic kidney disease	5 (20.8%)
Chronic obstructive pulmonary disease	5 (20.8%)
Insulin-dependent diabetes mellitus	2 (8.3%)
Atrial fibrillation	14 (58.3%)
– Chronic	5 (20.8%)
– Paroxysmal	9 (37.5%)
Pacemaker/ICD	0 (0%)
Endocarditis	3 (12.5%)

**Table 2:** Echocardiographic Data.

Parameter	Preoperative	Postoperative	*p*-Value
LVEF (%)	51.3 ± 11.2	51.9 ± 10.7	0.67
Aortic MPG (mmHg)	51.3 ± 23.0	6.7 ± 1.7	<0.001
Mitral MPG (mmHg)	19.3 ± 26.7	4.0 ± 1.4	<0.001
Aortic stenosis, *n* (%)	19 (79%)	0	—
Aortic regurgitation, *n* (%)	7 (29%)	0	—
Mitral regurgitation, *n* (%)	20 (83%)	0	—
Mitral stenosis, *n* (%)	6 (25%)	0	—
Postoperative aortic valve leakage, *n* (%)	—	0	—
Postoperative mitral valve leakage, *n* (%)	—	0	—

**Table 3:** Intraoperative Data.

Variables	
Operative time, min	252.6 ± 47.58
Aortic cross-clamp time, min	109.6 ± 26.8
Cardiopulmonary bypass time, min	159.6 ± 33.8
Transfusion of Red blood cell units	0.3 ± 0.71
Redo surgery	0 (0%)
MAZE procedure	0 (0%)
Left atrial appendage occlusion	4 (16.7%)

**Table 4:** Implanted Valve Prostheses and Sizes.

Valve	Procedure Type	Sample Size	Valve Types	Mean size ± SD	Range
Aortic	replacement	24 cases	- Perceval: 12 cases (50%) - Hancock: 8 cases (33.3%) - Inspiris: 2 cases (8.3%) - Epic: 2 cases (8.3%)	24.2 ± 1.0 mm	23–25 mm
Mitral	replacement	13 cases	- Hancock: 9 cases (69.2%) - Epic: 3 cases (23.1%) - Inspiris: 1 case (7.7%)	28.2 ± 1.6 mm	27–31 mm
	repair	11 cases	- Memo 4D™ mitral annuloplasty: 11 cases (100%)	33.5 ± 2.0 mm	32–36 mm

Perceval (Corcym SRL, Saluggia, Italy, previously LivaNova), Epic (St. Jude Medical/Abbott, St. Paul, MN, USA), Hancock II (Medtronic, Minneapolis, MN, USA), and Inspiris (Edwards Lifesciences, Irvine, CA, USA), Memo 4D™ mitral annuloplasty (Corcym, Saluggia, Italy).

**Table 5:** Postoperative Data.

Variables	
Hospital Stay (Days) [median (IQR)]	11 (9–15)
ICU Stay (Days) [median (IQR)]	2 (1–3)
New-onset AF	0 (0%)
Atrioventricular Block Grade III	2 (8.4%)
Pacemaker implantation	2 (8.4%)
Myocardial Infarction	0 (0%)
Conversion to Conventional Surgery	0 (0%)
Reoperation Valve Replacement	0 (0%)
Structural Valve Deterioration	0 (0%)
Pneumothorax	0 (0%)
Respiratory Insufficiency	1 (4.2%)
Re-thoracotomy for major bleeding	1 (4.2%)
Vascular Injury	0 (0%)
Lower limb Ischemia	0 (0%)
Wound-Healing Disorder	0 (0%)
Vascular Occlusion/Embolism	0 (0%)
Postoperative Dialysis	2 (8.4%)
intracranial hemorrhage	0 (0%)
Stroke	0 (0%)
Seizure	0 (0%)
Delirium	2 (8.4%)
Neurological Deficits	0 (0%)
Sepsis	0 (0%)
Resuscitation	0 (0%)
Impella	0 (0%)
ECMO/right ventricle dysfunction	0 (0%)
Extracorporeal Life Support	0 (0%)
In-hospital Mortality	1 (4.2%)
30-Day Mortality	1 (4.2%)

## Data Availability

Data is provided within the manuscript.
